# Assessment of Bioactive Phytochemicals and Utilization of *Rosa canina* Fruit Extract as a Novel Natural Antioxidant for Mayonnaise

**DOI:** 10.3390/molecules28083350

**Published:** 2023-04-10

**Authors:** Osama I. A. Soltan, Hanaa S. S. Gazwi, Amany E. Ragab, Abdullah S. M. Aljohani, Ibrahim M. El-Ashmawy, Gaber El-Saber Batiha, Amin A. Hafiz, Sanaa M. Abdel-Hameed

**Affiliations:** 1 Department of Food Science, Faculty of Agriculture, Minia University, El-Minia 61519, Egyptsanaa.mohamed@minia.edu.eg (S.M.A.-H.); 2 Department of Agricultural Chemistry, Faculty of Agriculture, Minia University, El-Minia 61519, Egypt; 3 Department of Pharmacognosy, Faculty of Pharmacy, Tanta University, Tanta 1527, Egypt; amany.ragab@pharm.tanta.edu.eg; 4 Department of Veterinary Medicine, College of Agriculture and Veterinary Medicine, Qassim University, Buraydah 52571, Saudi Arabia; a.alashmawi@qu.edu.sa; 5 Pharmacology Department, Faculty of Veterinary Medicine, Alexandria University, Alexandria 21521, Egypt; 6 Department of Pharmacology and Therapeutics, Faculty of Veterinary Medicine, Damanhour University, Damanhour 22511, Egypt; gaberbatiha@gmail.com; 7 Department of Clinical Nutrition, Faculty of Applied Medical Sciences, Umm Al-Qura University, Makka Al-Mukarama 21961, Saudi Arabia; aahafiz@uqu.edu.sa

**Keywords:** *Rosa canina* fruit extract, GC–MS, HLPC, shelf life, mayonnaise, sensory evaluation

## Abstract

The oxidation of food emulsions causes rancidity, which reduces their shelf life. To prevent rancidity, synthetic antioxidants are widely used in the food industry. However, due to their potential health risks, researchers are exploring natural alternatives. This study aimed to investigate whether *Rosa canina* fruit extract (RCFE) could be used as a natural antioxidant to extend the shelf life of mayonnaise. Mayonnaise containing varying concentrations of RCFE [0.125% (T1), 0.25% (T2), 0.50% (T3), 0.75% (T4)] was compared to a mayonnaise control sample (C1) and a mayonnaise sample containing 0.02% BHT (C2) for 60 days of storage at 4 °C. RCFE was found to have high levels of total phenols content (52.06 ± 1.14 mg GAE g^−1^), total flavonoids content (26.31 ± 1.03 mg QE g^−1^), and free radical scavenging activity. The GC–MS analysis of RCFE revealed 39 different peaks, whereas the HPLC analysis showed the presence of 13 polyphenolic compounds in RCFE. The pH values of T2, T3, and T4 mayonnaise samples substantially declined as storage progressed; however, the reduction was less than that of C1 and C2. After 60 days, mayonnaise samples T2, T3, and T4 had greatly reduced peroxide and free fatty acid levels compared to C1 and C2. The mayonnaise enriched with RCFE (T3 and T4) had the most potent antioxidative ability and the lowest value of lipid hydroperoxides (peroxide value, POV) and the lowest value of thiobarbituric-acid-reactive substances (TBARS). The sensory evaluation revealed that the T3 sample exhibited the highest overall acceptability. In conclusion, this study recommends that RCFE could be used as a natural preservative to enhance the shelf life of functional foods.

## 1. Introduction

There is a rising trend in the food industry to utilize plant extracts as antimicrobials and as an alternative to synthetic antioxidants to prevent lipid oxidation, fight foodborne pathogens, and extend the shelf life of food [1]. Recent research has focused on the extraordinary health benefits of polyphenols on oxidative stressors linked to diseases such as cardiovascular diseases, cellular aging, and cancer [2]. Medicinal plants are an indispensable source for bioactive natural products, as recommended by the World Health Organization [3]. Evidently, medicinal plants have attracted the researchers’ interest to learn more about their efficiency, properties, and safety [4]. Amongst the bioactive chemicals from plants, natural antioxidants intrigued the researchers due to their involvement in treating different diseases as well as in industries.

Natural antioxidants have the potential to delay or prevent oxidative damage of fats. Thus, their incorporation in fat-based food is an effective method for avoiding the deterioration in the product quality mainly caused by reactive oxygen species (ROS) attack [5]. Natural antioxidants act as preservatives if they limit lipid oxidation by protecting target lipids from oxidation initiators or via reducing oxidation spread, a process known as “chain-breaking” [6]. In recent years, food enrichment has appeared to be a simple and inexpensive method to improve nutritional quality.

Plants have been the primary target of the worldwide search for novel functional food sources [7]. The dog rose, *Rosa canina* (Rosaceae), is a well-known thorny shrub with fragrant pink or white flowers. Other common names include rose hip and briar rose. The respective genus is found worldwide, such as in Asia, North America, and the Middle East [8]. Rosehip fruits can be processed into healthy, aromatic, and functional products, such as rosehip tea, nectar, syrups, jam, marmalade, pistil, extruded snacks, and rosehip vinegar [9,10]. The extracts of this plant’s pseudo-fruits are characterized by their high content of bioactive phytochemicals, which include carotenoids, tocopherols, vitamin C, polyphenols, and flavonoids that have the potential to act as natural antioxidants [5,10,11]. They also have preventive and curative effects for diabetes, memory dysfunction, and cancer. Furthermore, these extracts have been widely employed as antimicrobial, antinociceptive, and anti-inflammatory agents [9].

To reduce rancidity as well as delay toxic oxidation product formation, antioxidants are included in foods, especially lipid-containing foods [12]. The potential carcinogenic consequences of synthetic antioxidants such as BHT and BHA cause concern among consumers. These preservatives, which include sorbates, formaldehyde, nitrates, sulfites, benzoates, and others, can cause death and have been linked to symptoms such as abdominal pain, anaphylactic shock, and asthma [13]. As a result, there has been a rising global trend toward consumer demand for natural antioxidants [14].

Quail eggs are a valuable source of proteins, fats, vitamins, minerals, and other essential micronutrients. Their nutritional quality is greater than chicken eggs. They have not been known to cause any allergies. Hence, they are recommended for regular consumption to help fight against many diseases [15]. Mayonnaise is currently the most popular sauce worldwide, so we used it as a model. Because emulsified matrices are so difficult to create, few studies have been completed to examine how polyphenols from plant residues affect them. Mayonnaise is an oil-in-water (o/w) food emulsion, which means that lipid oxidation usually starts at the oil–water interface and moves to the oil phase as the food sits [16].

This study aimed to determine the chemical composition of RCFE and its protective effect in inhibiting oxidative deterioration and increasing the nutritional value and shelf life of mayonnaise prepared using quail eggs. This study is the first of its kind on this plant extract and serves as the basis for an efficient and cost-effective mayonnaise enrichment method, as well as contributing to the value-added use of plant residues.

## 2. Results and Discussion

### 2.1. Phytochemicals Analysis

Table 1 displays the results of the phytochemical screening. As indicated in Table 1, the RCFE contains flavonoids, phenols, saponins, tannins, terpenoids, alkaloids, anthocyanins, sterols, glycosides, and fatty acids. Saponins, steroids, alkaloids, triterpenoids, and tannins are the other primary plant elements that function primarily as antioxidants or free radical scavengers and anti-inflammatory agents (Table 1) [6]. Tannins (high-molecular-weight phenols) can quench free radicals, which depends on the molecular weight, aromatic rings, and kind of substitution of the hydroxyl group [2].

### 2.2. Total Phenolics, Flavonoids Content, and Antioxidant Activity

Spectrophotometric analysis was used to determine the polyphenolic and flavonoid concentrations in the RCFE (Table 2). The *Rosa canina* extract contents of total phenolics and flavonoids were 52.06 ± 1.14 mg GAE/g extract and 26.31 ± 1.03 mg QE/g extract, respectively (Table 2).

Our findings agree with previous studies on the total phenolic content of *Rosa canina* L. fruits from different regions [11]. Polyphenols have a high scavenging activity for ROS, and their hydrogen-donating properties are involved in the suppression of lipid oxidation [1]. Samec et al. [2] related the antioxidant activities to polyphenols, which scavenge singlet oxygen, lipid peroxyl radicals, and hydroxyl, in addition to preventing lipid oxidation. Flavonoids are one of the most common types of natural constituents, and they have antioxidant properties by chelating metal ions and scavenging free radicals [17].

Phenolic molecules such as flavonoids, anthocyanins, and phenolic acids have many associated biological activities, such as anti-inflammatory, antioxidant, and anticancer properties [18]. In this study, the DPPH and FRAP assays were utilized for measuring the RCFE antioxidant activity (Table 2). The IC_50_ value for the RCFE was 89.16 ± 2.76 μg/mL and for Trolox was 110.34 ± 0.71 μg/mL.

According to the results, the RCFE can act as a free radical scavenger due to its phenolic components. Ousaaid et al. [11] found that, when antioxidants react with fatty acid peroxy radicals, the immobilized electrons around the aromatic ring move away from the ring and stabilize the phenoxy radicals that are formed. Gazwi [19] indicated that the amount of phenolics strongly influences the free radical scavenging activity of DPPH. In our study, the RCFE was effective at scavenging DPPH free radicals.

Additionally, a direct correlation exists between the total phenolics and FRAP. The FRAP value of the RCFE was 96.81 ± 3.07 (μM Trolox/mg extract), as depicted in Table 1.

### 2.3. GC–MS Analyses

**Figure 1:**
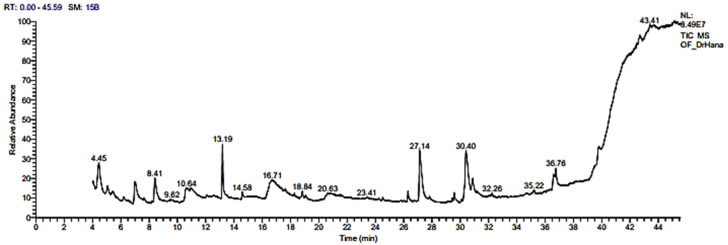
GC–MS analyses of the RCFE.

GC–MS analysis of the RCFE was performed to evaluate its phytochemical components. The results indicated that 39 different phytochemicals were identified by the GC–MS analysis (Table 3 and Figure 1). The major components identified were hexadecanoic acid (12.72%), 9,12-Octadecadienoyl chloride, (Z, Z)- (10.45%), glycerol 1,2-diacetate (9.00%), maltose (7.58%), 2-methylcyclopentanone (6.36%), cyclohexanamine, N-3-butenyl-N-methyl- (5.06%), hexadecenoic Acid,1-(hydroxymethyl)-1,2-ethanediyl ester (4.55%), oleic acid (3.41%), hexadecanoic acid, 2,3-dihydroxypropyl ester (3.36%), 7,8-epoxylanostan-11-ol, and 3-acetoxy- (3.29%). These components have been studied previously for their therapeutic and biological properties (Table 4). Other components’ various applications have also been revealed. For example, glycerol 1,2-diacetate is used as a flavoring agent and a food additive, whereas α-lactose and α-D-glucopyranose are employed as food sweetening agents (Table 4).

### 2.4. HPLC Analysis

**Figure 2:**
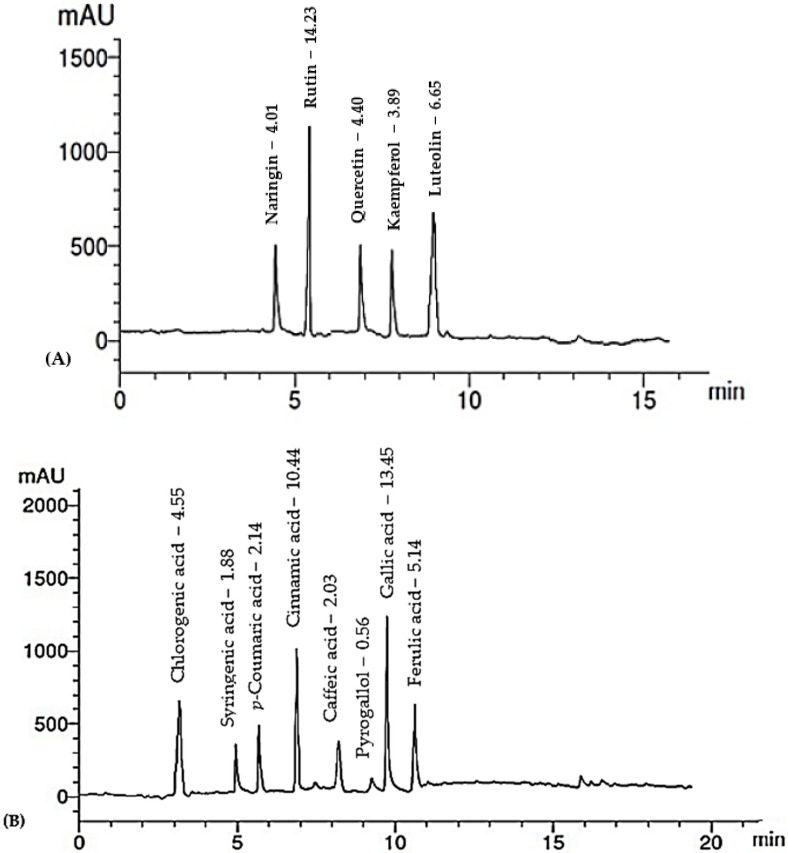
HPLC chromatogram of identified flavonoids (**A**) and phenolic acids (**B**) in the RCFE.

The HPLC analysis of the RCFE revealed thirteen polyphenolic compounds, including five flavonoids and eight phenolic acids, as presented in Table 5 and Figure 2. The compounds were confirmed by comparing them with authentic samples analyzed using the same conditions. Rutin and luteolin were the major flavonoids identified at concentrations of 14.23 and 6.65 μg/mg of the RCFE, respectively. Gallic acid was found to be the predominant phenolic component in the extract (13.45 μg/mg), followed by cinnamic acid (10.44 μg/mg) and ferulic acid (5.14 μg/mg), as shown in Figure 2 and Table 5.

### 2.5. Quality Evaluation of Quail Egg Mayonnaise Enriched with RCFE

#### 2.5.1. Changes in pH Values

Table 6 shows the changes in pH of all mayonnaise samples throughout 60-day storage at 4 °C. The results revealed that all samples’ pH values decreased throughout the storage period. Mayonnaise samples containing different amounts of the RCFE (T1, T2, T3, and T4) show a lower reduction in the pH compared to the control mayonnaise (C1) and the mayonnaise containing BHT (C2). When the concentration of the RCFE was increased in the mayonnaise sample, a lesser decline in the pH of the mayonnaise was noticed with storage periods. Mayonnaise containing BHT (C1) had a lower pH drop than the control sample (C1). After 60 days, the pH values of mayonnaise samples C1, C2, T1, T2, T3, and T4 were 2.76, 3.85, 3.79, 3.89, 3.91, and 3.96, respectively.

The decrease in the pH can be related to a rise in the acidity caused by triglyceride hydrolysis and, thus, an increase in free fatty acid content [20]. As more microorganisms grow in the stored mayonnaise, they produce more organic acids, resulting in a decrease in pH. According to Triawati et al. [21], the high concentration of organic acids in mayonnaise alters its appearance and texture.

#### 2.5.2. Changes in the Acid Value

The generation of acid value (free fatty acids) could determine mayonnaise rancidity, and the reaction of unsaturated fats with moisture increases the amount of acid value by hydrolysis of the lipids. The acid value rises with the oxidation rate because a high oxidation rate results in a high concentration of long-chain free fatty acids. Table 7 depicts the rise in the acid value as the storage time progressed. During storage, the acid value was elevated in all the mayonnaise samples; nonetheless, the elevation was less noticeable in the samples containing BHT and RCFE. The lipids isolated from the mayonnaise control sample demonstrated that it was highly susceptible to triglyceride hydrolysis during storage, resulting in a more remarkable synthesis of acid value. After 60 days, the acid value detected from the control sample (C1) was 6.54 ± 0.05 mg g^−1^; however, the acid value contents of samples C2, T1, T2, T3, and T4 were 3.17 ± 0.08, 3.72 ± 0.04, 3.17 ± 0.07, 3.03 ± 0.01, and 2.99 ± 0.02 mg g^−1^, respectively. The mayonnaise containing different concentrations of the RCFE and the synthetic antioxidant BHT was substantially different from the control sample of mayonnaise. The RCFE inhibited triglyceride hydrolysis more effectively than BHT. There may be some correlation between the total plate count (TPC), pH variations, and acidity levels in all samples throughout storage, as confirmed by Gani et al. [22]. When samples were kept at 4 °C, TPC showed a statistically significant (*p* ≤ 0.05) association, with both decreasing pH and increasing acidity. Al Akeel et al. [4] showed that the RCFE had effective antibacterial activities. This finding suggests that the bioactive components in the RCFE-enriched mayonnaise sample may contribute to preserving the microbiome during storage.

#### 2.5.3. Oxidative Stability of Mayonnaise

1. Changes in peroxide value

At the initial oxidation stage, peroxide value (POV), which assesses the quantity of primary oxidation, is a sign of the beginning of autoxidation or oxidative rancidity [23]. POV in all mayonnaise samples had elevated with storage time, reaching their greatest levels on day 60. The high concentration of phenolics in the RCFE had a protective impact on mayonnaise POV during storage. By preventing lipid oxidation, phenolic compounds can slow the rise in the peroxide levels. Mayonnaise samples containing 0.5% and 0.75% RCEF had a reduced POV value of 6.96 and 4.74 meq kg^−1^ oil, respectively, after 60 days (Table 8). Mayonnaise treated with the RCFE (0.25%, 0.5%, and 0.75%) showed an increased POV less than the mayonnaise sample treated with the synthetic antioxidant BHT and the control sample (Table 8). After 60 days in storage, the control sample, which had the lowest oxidative stability, shows the greatest peroxide readings (Table 8).

Pro-oxidants create highly reactive alkoxyl and peroxyl radicals by breaking down lipid hydroperoxides (ROOH). They form free lipid radicals by reacting with unsaturated fatty acids in droplets or at the O/W contact. In this way, these lipid radicals continue the oxidation chain process by reacting with nearby lipids. Antioxidants have been found to provide a hydrogen atom to free radicals, breaking the chain reaction’s propagation during lipid oxidation [24]. As the concentration of the RCFE was raised, the mayonnaise samples’ oxidative stability improved.

Similar observations were found by Alizadeh et al. [25] for the mayonnaise enriched with *Ferulago angulata* extract, rosemary essential oil, and tocopherol during its shelf life and Nour [26] for mayonnaise boosted with carotenoids from sea buckthorn pomace under refrigeration storage.

2. Thiobarbituric-acid-reactive species (TBARS) values

The TBARS analysis is used to measure the secondary oxidation byproducts formed during lipid oxidation, especially malondialdehyde (MDA), that might contribute off-flavor to oxidized fat [12]. In addition, Yang et al. [14] observed in their study that, in the oxidation stage, the decomposition of peroxides into lower-molecular-weight components such as malonaldehyde occurs. In our study, the control (C1) samples’ TBARS values elevated with storage time. These results suggested that BHT (C2) efficiently reduced lipid oxidation, while the RCFE substantially reduced it (T3 and T4), as shown in Table 9. At the end of storage, the TBARS values of mayonnaise containing 0.50% RCFE (T3) and 0.75% RCFE (T4) were lower (*p* ≤ 0.05) (0.676 and 0.631 mg MAD kg^−1^ of sample, respectively) than in the control sample (C1) as well as BHT (C2) (1.516 and 0.722 mg MAD kg^−1^, respectively), as shown in Table 9. This result can be attributed to the phenolic chemicals in the extract, acting as electron or hydrogen donors to replace the radicals in the reaction, thereby preventing the production of hydroperoxides as well as subsequent products or delayed unsaturated fatty acid oxidation [27]. These findings demonstrated that the antioxidants inhibited lipid oxidation during and soon following enrichment, confirming Rasmy et al.’s findings [22]. Yang et al. [14] and Aleman et al. [23] discovered a similarly strong correlation between phenolic content and plant extract antioxidant activity. It could be demonstrated that polyphenols prevented the oxidation of lipids during storage.

3. Microbiological analysis

**Figure 3:**
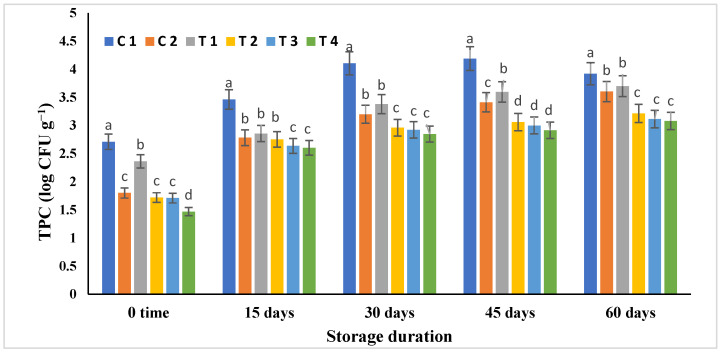
Microbial analysis of mayonnaise samples enriched with the RCFE during storage. Means with the same letter in columns are not statistically significant (*p* ≤ 0.05), whereas means sharing a different letter in columns of the table are significantly statistical (*p* ≤ 0.05). TPC, total plate count; C1, mayonnaise with no antioxidants; C2, mayonnaise treated by 0.02% BHT; T1, T2, T3, T4: mayonnaise treated by 0.125, 0.25, 0.5, and 0.75 % ethanolic *Rosa canina* extract, respectively.

Figure 3 shows the results of a microbial analysis of all mayonnaise treatments during storage at 4 °C for 60 days. Total plate count (TPC) for all mayonnaise samples stored at 4 °C substantially increased (*p* ≤ 0.05) over time (Figure 3). Possible sources of microbial contamination include the use of contaminated eggs, utensils, and equipment, as well as environmental factors such as air quality, temperature, and relative humidity and storage conditions [16]. Mayonnaise samples containing BHT and RCFE had considerably diminished (*p* ≤ 0.05) TPC compared to the mayonnaise control sample through all storage periods. At time zero, the TPC of all mayonnaise samples was low, except for the C1 and T1 samples (2.71 and 2.36 log CFU g^−1^, respectively). With storage time progression, the TPC substantially elevated (*p* ≤ 0.05) in all mayonnaise samples. After 60 days of storage, the lowest TPC (3.08 and 3.11 log CFU g^−1^) was detected in both the T4 and T3, whereas the highest TPC (3.92 log CFU g^−1^) was found in the control (C1).

The effect of soluble and undissociated acetic acid in the oil stage may explain the decrease in total bacterial counts on the first day of storage. Organic acids have antimicrobial effects because they lower the pH of their surroundings, accumulate anions, disrupt membrane transport and permeability, and/or lower the pH of the intracellular environment by dissociating acid from hydrogen ions. The TPC in control increased after the storage period, most likely due to the evolution of acid-tolerant microorganisms [28].

The inclusion of natural extracts in mayonnaise functions as an antibacterial agent, which inhibits the growth of bacteria and thus extends the shelf life of the final products [29].

#### 2.5.4. Sensory Evaluation

All mayonnaise samples’ sensory evaluation, including taste, color, mouth feel, odor, and overall acceptability at 4 °C for a period of 60 days, were evaluated and displayed in Table 10, Table 11 and Table 12, respectively. Storage period and the RCFE concentrations greatly affected the evaluation of sensory qualities, such as color, taste, overall acceptability, and mouthfeel.

Since mayonnaise is a lipid-rich emulsion that oxidizes as storage time increases, these findings indicate that mayonnaise samples received the highest score at day zero as well as the lowest score by the conclusion of storage for sensory evaluation. The quality of mayonnaise decreased as storage time increased as the oxidation rate peaked. The RCFE addition to mayonnaise slows the rate of oxidation. Panelists rated the control sample (C1) as the least favorable at the end of 60 days, which appears to be due to triglyceride breakdown. The mayonnaise sample enriched with 0.5% RCFE (T3) was the panelists’ favorite and scored the highest throughout storage, eventually outperforming the mayonnaise sample treated with BHT (C2). T1 was observed to have a low score compared to T2, T3, and T4.

Nour [26] discovered comparable outcomes during the refrigeration storage of mayonnaise supplemented with carotenoids extracted from sea buckthorn pomace.

The polyphenols, natural antioxidants present in *Rosa canina* extract, can protect against lipid oxidation and preserve the quality and flavor of the enriched sample from alteration during storage. The results were similar to those of Ochoa-Velasco and Guerrero-Beltran [7].

#### 2.5.5. Mayonnaise Samples’ Color Characteristics Enriched with RCFE during Storage

**Figure 4:**
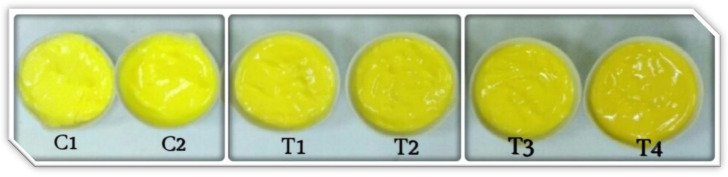
The photographs of control and RCFE-enriched mayonnaise samples. C1, mayonnaise with no antioxidants; C2, mayonnaise treated by 0.02% BHT; T1, T2, T3, T4: mayonnaise treated by 0.125, 0.25, 0.5, and 0.75% ethanolic *Rosa canina* extract, respectively.

**Figure 5:**
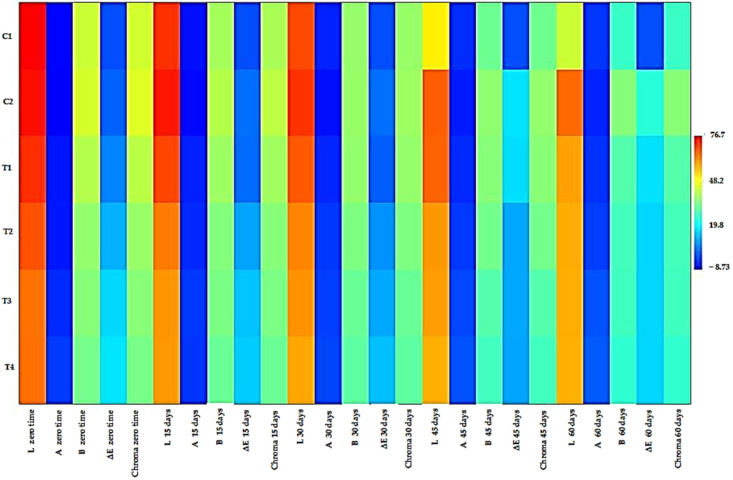
Correlation of the color characteristics of mayonnaise samples enriched with RCFE during storage.

The color of mayonnaise is crucial in determining consumer preference. Mayonnaise’s bright yellow color is typically associated with a high egg content, implying additional nutritional and biological benefits and flavor. The supplementation of mayonnaise with unconventional additives that differ from those used in a standard recipe could lead to physical and chemical changes that impact the color of the final products. Figure 4 and Figure 5 and Table 13 and Table 14 show the color parameters (L, a, b, chroma, and ΔE) of the mayonnaise samples during storage. The changes in the lightness (L*), yellowness (b*), and redness (a*) values in Table 13 show that the yellowness (b*) as well as the lightness (L*) of all mayonnaise samples diminished, whereas the redness (a*) gradually increased. At the onset of the storage interval (day 0), the L* value of the control mayonnaise (C1) was 76.73 and decreased to 42.53 by storage (day 60), while the L* value of the mayonnaise enriched with BHT (C2) was 76.27 at the beginning of the storage period (day 0) and declined to 64.83 at the end of the storage period (day 60). The L* values of mayonnaise with RCFE (T1, T2, T3, and T4) were 71.67, 67.67, 64.10, and 64.20, respectively, at day 0 and declined to 58.70, 57.57, 57.37, and 57.03, respectively, at day 60. Overall, these findings show that the addition of the RCFE did not affect the visual appearance of the emulsion. The results in Table 13 revealed that the control (C1) and RCFE-enriched mayonnaise samples’ a* values dramatically changed for 60 days. There were substantial differences between the redness values (a*) of the control sample (C1) and mayonnaise samples enhanced with RCFE (T2, T3, and T4). When compared to the control sample (C1), mayonnaise enriched with 0.25, 0.5, and 0.75% RCFE (T2, T3, and T4, respectively) had significantly higher yellowness (b*) values. At the end of storage, the highest L*, a*, and b* values were observed in C2, T4, and C2, respectively, while the lowest L*, b*, and a* values were found in C1, C2, and T4, respectively.

Non-enzymatic browning reactions using carbonyl compounds formed during lipid oxidation as substrates, besides polymerization of brown oxypolymers formed from derivatives of lipid oxidation, were found to contribute to the darkening of mayonnaise samples during storage [30].

Based on the above results, it could be observed that the color parameters (L*, a*, and b*) were significantly changed during storage for all mayonnaise samples. Hence, significant variations in the values of ΔE were found in some cases (Table 14). At zero time, the highest color changes (ΔE) were observed in the case of T4 (16.95), followed by T3 (15.38), T2 (11.15), and T1 (6.04). Conversely, the mayonnaise enriched with BHT (C2) recorded the lowest color changes (2.10). At the end of storage (60 days), the highest color changes (ΔE) were observed in C2 (24.04), followed by T1 (16.49). In contrast, the RCFE-enriched mayonnaise samples T2, T3, and T4 recorded nearly the same color changes (15.14–15.19) compared to the control (C1). Despite these changes, all enriched mayonnaise samples had an acceptable color. It is well-understood that preferred colors are most similar to the original color of control samples [31,32].

Table 14 shows that all the mayonnaise samples’ chroma values (a measure of color saturation and intensity) declined noticeably during storage as the yellowness (b-value) and redness (a-value) outcomes shifted. It could also be observed that the incorporation of the RCFE up to 0.75% level caused a significant decrease in chroma values. This decrease was proportional to the amount of the RCFE used to enrich the mayonnaise samples. The chroma values were significantly decreased as the incorporated levels of RCFE into mayonnaise samples increased.

## 3. Materials and Methods

### 3.1. Ethanolic Rosa canina Fruits Extract (RCEF) Preparation

After washing the fruits, they were dried at 40 °C in a forced air oven and powdered in a grinder to obtain 40-mesh-size powder before extraction. The dried powder of RCFE (0.5 kg) was soaked in aqueous ethanol (80%) at a ratio (1:10 *w/v*) with shaking overnight at room temperature, followed by filtration through Whatman paper (No.1). At 45 °C, evaporation of the filtrate was carried out utilizing a rotary evaporator under vacuum to obtain a brown residue (yield 15% *w*/*w*, as calculated) [33]. The extract residue was kept at −20 °C until further usage.

### 3.2. Phytochemicals Analysis of RCEF

A preliminary phytochemical analysis of the main constituents in the extract, such as saponins, alkaloids, steroids, tannins, and triterpenoids, was carried out following Ref. [6].

### 3.3. Total Phenolic and Total Flavonoid Contents

Total flavonoid (TFC) as well as phenolic (TPC) contents were estimated using the methods described by Farhadi et al. [1] and Singleton and Rossi [34], respectively. The TFC was assessed as milligrams of quercetin equivalents per gram of the RCFE (mg QE g ^−1^) and the TPC was expressed as milligrams of gallic acid equivalents per gram of the RCFE (mg GAE g^−1^).

### 3.4. Antioxidant Activity Assay

The RCFE antioxidant activity was measured by their capacity to get rid of DPPH (2,2-diphenyl-1-picrylhydrazyl) free radicals using a previously described method (Lee et al. [35]), and the assay of ferric-reducing antioxidant power (FRAP) was assessed based on the method of Adedapo et al. [36].

### 3.5. GC–MS Analysis

The RCFE was subjected to GC–MS analysis using a Trace GC1310-ISQ mass spectrometer (Thermo Scientific, Austin, TX, USA) equipped with a direct capillary column TG-5MS (30 mm × 0.25 mm × 0.25 μm film thickness, Thermo Scientific, Austin, TX, USA). The column’s oven was initially maintained at 50 °C and then gradually heated to 200 °C at a rate of 7 °C/min, held for 2 min, and further heated to 290 °C at a rate of 15 °C/min and held for 2 min. The injector temperature was set at 260 °C, and helium was used as the carrier gas with a constant flow rate of 1 mL/min. An AS3000 autosampler and GC in split mode were used to inject 1 μL of the diluted sample after a 4 min solvent delay. The mass spectra were obtained in a full scan mode at 70 eV ionization voltage across the *m/z* 50–650 range. The transfer line and ion source temperatures were adjusted to 250 and 270 degrees, respectively. The components were identified by comparing their mass spectra retention times to those in the NIST 11 and WILEY 09 mass spectral databases.

### 3.6. HPLC Analysis

The HPLC system (Agilent 1100; Santa Clara, CA, USA) was used to determine the phenolic and flavonoid components of the RCFE. The extract was injected with a volume of 25 μL, and the procedure was performed as described previously [37]. To identify the extract’s phenolic components, a C18 column (125 × 4.60 mm, particle size 5 μm) and a UV/Vis detector at a wavelength of 250 nm were employed. The Agilent Chem Station was utilized to obtain and analyze chromatograms. A mobile gradient phase consisting of methanol [A] and acetic acid in water (1:25) [B] was utilized to completely separate the phenolic acid components. The gradient program began at 100% B, stayed for the first three min, and then shifted to 5 min of 50% eluent A, 2 min of 80% A, 5 min of 50% A. The detection wavelength was set at 250 nm. The same HPLC system and a C18 column (250 × 4.6 mm, 5 μm) were used to identify the flavonoid components in the extract. The UV/Vis detector was set at a wavelength of 360 nm. Acetonitrile (A) and 0.2% (*v/v*) aqueous formic acid (B) were utilized as the mobile phase, and an isocratic elution procedure (70:30) was used.

### 3.7. Mayonnaise Preparation

The preparation of mayonnaise samples was completed per the method of Khalid et al. [38] with some modifications. The mayonnaise formula was developed by mixing the following components in the following percentage (*w/w*): sunflower oil (70%), whole quail egg (20%), vinegar (6%), salt (1%), and sugar (3%). The mayonnaise control samples were prepared by blending eggs and vinegar together followed by all the other components utilizing an electric blender (Bosch hand blender Clever Mixx, Stuttgart, Germany). Then, the oil was gradually added to the aqueous mixture with a steady rise in the speed of blending when the mayonnaise mass began to thicken. Both control mayonnaise samples C1 and C2 were produced without adding the RCFE. Nevertheless, control mayonnaise sample C2 was prepared with the addition of 0.02% (0.2 mg/g) BHT. The treated mayonnaise samples were produced in a similar way to C1 with further addition of the RCFE extract at four different concentrations of 0.125% (1.25 mg/g), 0.25% (2.5 mg/g), 0.50% (5.0 mg/g), and 0.75% (7.5 mg/g) and coded T1, T2, T3, and T4, respectively. The obtained mayonnaise samples were packed and kept at 4 °C until analyzed at zero time (after two days) and during storage every 15 days.

### 3.8. Shelf-Life Study of Mayonnaise Fortified with RCFE

#### 3.8.1. Peroxide Values (POV) and Thiobarbituric-Acid-Reactive Species (TBARS) Values

Peroxide and TBARS levels were analyzed to determine the RCFE’s oxidative stability. Mayonnaise PV was measured utilizing the method of Siwach et al. [12]. The supernatant was collected when 0.15 g of mayonnaise was centrifuged (at 2000 r/min for 5 min) with 1.5 mL of an isooctane/isopropanol combination (2:1, *v/v*). In the absence of light for 20 min, 0.5 mL of the supernatant was combined with 20 mL of KSCN (3.94 mol/L), 20 mL of FeSO_4_ (0.072 mol/L), and 3 mL of methanol/*n*-butanol combination (2:1, *v/v*). The reaction mixture absorbance was determined at a wavelength of 510 nm. The levels of PV were measured in mayonnaise samples taken at 0, 15, 30, 45, and 60 storage days.

The values of TBARS in mayonnaise samples were measured using the technique Ye et al. [39] reported with minor modifications. Further, 0.3 g of sample of mayonnaise, 2.5 mL of thiobarbituric acid (1%) solution, and 1 mL of trichloroacetic acid (TCA) solution (10%) were thoroughly mixed before boiling for 30 min. Then, 0.5 mL of chloroform was added to the mixture and shaken thoroughly before centrifugation for 15 min (6000 r/min) to separate the supernatant. At a wavelength of 532 nm, the absorbance of the supernatant was determined. Mayonnaise samples were collected at 0, 15, 30, 45, and 60 days of storage, and the TBARS levels were analyzed.

##### 3.8.2. pH and Acid Values

The method used to determine the pH values of mayonnaise samples was modified from the method of Santipanichwong et al. [40]. Mayonnaise samples (2 g) and 18 mL of water were mixed and evenly dispersed with a vortex shaker, whereas the mixture pH was determined using a pH meter (PB-10, Sartorius, Gottingen, Germany).

Acid values of the mayonnaise samples were estimated by the methods of AOCS [41]. The following equation calculates the acid value, which is the potassium hydroxide (KOH) number of milligrams required to neutralize the acids utilizing 1 g of fatty sample:

Acid value = (Titration mL × 56.1 × N)/the collected sample weight

where N is KOH normality (0.1 N) and 56.1 is KOH molecular weight.

#### 3.8.3. Microbial Analysis

Samples of mayonnaise were diluted serially 10-fold. Next, 100 µL of the diluted mayonnaise samples were poured onto an agar counting plate medium before incubation for 48 h at 37 °C and then bacterial counting [7].

#### 3.8.4. Sensory Evaluation

Mayonnaise samples’ sensory characteristics for different parameters were conducted every 15 days by twenty panelists (ten men and ten women), each seated under white light in a separate well-ventilated room. Among the panelists were faculty members as well as technical staff from the Department of Food Technology. Even though they were regular mayonnaise consumers, we explained the procedure as well as the conditions of tasting. Sensory evaluation was scored using a 9-point hedonic scale: 9: like greatly; 8: like very much; 7: like moderately; 6: like slightly; 5: neither dislike nor like; 4: dislike slightly; 3: dislike moderately; 2: dislike very much; 1: dislike greatly [7,12]. The mayonnaise samples were coded with random three-digit numbers and presented to the panelists in a random order [12].

#### 3.8.5. Color Measurement

The assessment of color characteristics of the mayonnaise samples was reported by Wang et al. [42]. The red–green value (a*), light–dark value (L*), and yellow–blue value (b*) of the mayonnaise samples were assayed utilizing a colorimeter (model color Tec-PCM, USA). Furthermore, the total chroma as well as color difference (ΔE) were determined by the equations:

ΔE = [(L − L_o_) ² + (a − a_o_) ² + (b − b_o_) ²] ½

Chroma = [(a² + b²)] ½(1)

where: L_o_, b_o_, and a_o_ were the L, b, and reference sample values, which is the control one.

### 3.9. Statistical Analysis

All data were presented as means ± standard deviation (SD), and data analysis was completed utilizing one-way ANOVA followed by Tukey’s test for multiple comparisons. Statistical significance was set at *p* < 0.05.

## 4. Conclusions

In conclusion, the enrichment of mayonnaise with phenolic- and flavonoid-rich extracts from plants such as *Rosa canina* could be an effective substitute to synthetic preservatives and antioxidants. The RCFE-enriched mayonnaise exhibited considerably enhanced oxidative stability, as shown by its increased antioxidant capacity and decreased primary and secondary oxidation products. The RCFE was more efficient than BHA at delaying the oxidation in mayonnaise and lengthening its shelf life. The inclusion of RCFE at a concentration of 0.50% (T3) during the production of mayonnaise was deemed the most acceptable. These results encourage the use and assessment of natural antioxidants and preservatives in food industries.

## Figures and Tables

**Table 1:** Qualitative phytochemicals analysis of the ethanolic extract of *Rosa canina* fruit (RCFE).

Test for	RCFE
Tannins	+
Anthocyanins	++
Saponins	+
Flavonoids	++
fatty acids	++
Phenols	++
Steroids	++
Alkaloids	++
Emodins	−
Glycosides	++
Terpenoids	++
Coumarins	−

(++) means highly positive; (+) means positive; (−) means negative activity.

**Table 2:** Total flavonoids and phenolics content as well as antioxidant properties of RCFE.

Components	Values *
Total phenolics (mg GAE/g extract)	52.06 ± 1.14
Flavonoids (mg QE/g extract)	26.31 ± 1.03
DPPH (IC_50_ μg/mL)	89.16 ± 2.76
FRAP (μM Trolox/mg extract)	96.81 ± 3.07

* Values are expressed as mean ± standard deviation (*n* = 3).

**Table 3:** GC–MS analyses of the RCFE.

NO.	RT	Chemical Name	MF	MW	Peak Area (%)
1	4.04	9-Octadecenamide	C_18_H_35_NO	281	0.72
2	4.45	2-Methylcyclopentanone	C_6_H_10_O	98	6.36
3	5.06	Cyclohexane carboxylic acid, 2-hydroxy-, ethyl ester	C_9_H_16_O_3_	172	1.70
4	5.43	11-Hydroxyundecanoic acid, lactone	C_11_H_20_O_2_	184	1.00
5	6.21	2-Myristynoyl pantetheine	C_25_H_44_N_2_O_5_S	484	0.99
6	7.00	Cyclohexanamine, N-3-butenyl-N-methyl-	C_11_H_21_N	167	5.06
7	7.63	Methyl 6-oxoheptanoate	C8H14O3	158	0.55
8	8.40	4H-Pyran-4-one, 2,3-dihydro-3,5-dihydroxy-6-methyl	C_6_H_8_O_4_	144	5.49
9	10.60	5-Hydroxymethylfurfural	C_6_H_6_O_3_	126	3.24
10	10.95	Melezitose	C_18_H_32_O_16_	504	0.80
11	11.05	α-D-Glucopyranoside,O-α-D-glucopyranosyl-α -D-fructofuranosyl	C_18_H_32_O_16_	504	0.76
12	12.08	D-Fructose, diethyl mercaptal, pentaacetate	C_20_H_32_O_10_S_2_	496	0.50
13	13.19	Glycerol 1,2-diacetate	C_7_H_12_O_5_	176	9.00
14	14.58	Undecanoic acid, 11-amino-	C_11_H_23_NO_2_	201	1.51
15	16.35	4-O-Hexopyranosylhexopyranose	C_12_H_22_O_11_	342	0.55
16	16.51	2-Aminoethanethiol hydrogen sulfate (ester)	C_2_H_7_NO_3_S_2_	157	2.57
17	16.66	Maltose	C_12_H_22_O_11_	342	7.58
18	17.64	1,4-Diacetyl-3-acetoxymethyl-2,5-methylene-l-rhamnitol	C_14_H_22_O_8_	318	0.80
19	18.27	1,3,5-Triazine-2,4-Diamine,6-Chloro-N-Ethyl	C_5_H_8_ClN_5_	173	0.61
20	18.84	1H-Cycloprop[e]azulen-7-ol, decahydro-1,1,7-trimethyl-4-methylene-, [1ar-(1aà,4aà,7á,7aá,7bà)]-	C_15_H_24_O	220	1.14
21	19.06	Phen-1,4-diol,2,3-dimethyl-5-trifluoromethyl-	C_9_H_9_F_3_O_2_	206	0.66
22	20.50	α-D-Glucopyranose, 4-O-α-D-galactopyranosyl-	C_12_H_22_O_11_	342	0.51
23	20.63	Tetraacetyl-d-xylonic nitrile	C_14_H_17_NO_9_	343	0.64
24	21.48	1H-Indol-5-Ol, 3-(2-Aminoethyl)-	C_10_H_12_N_2_O	176	0.25
25	26.30	Pentadecanoic acid, 14-methyl-, methyl Ester	C_17_H_34_O_2_	270	1.76
26	27.13	Hexadecanoic Acid	C_16_H_32_O_2_	256	12.72
27	27.82	9-Octadecenoic Acid (Z)-	C_18_H_34_O_2_	282	0.83
28	29.44	Linoleic acid ethyl ester	C_20_H_36_O_2_	308,	0.55
29	29.58	10-Octadecenoic acid, methyl ester	C_19_H_36_O_2_	296	1.55
30	30.10	Cyclopropanedodecanoic acid, 2-Octyl-, methyl ester	C_24_H_46_O_2_	366	0.49
31	30.40	9,12-Octadecadienoyl chloride, (Z, Z)-	C_18_H_31_ClO	298	10.45
32	30.85	Oleic Acid	C_18_H_34_O_2_	282	3.41
33	32.25	1-Heptatriacotanol	C_37_H_76_O	536	0.61
34	35.22	4-Hexyl-1-(7-methoxycarbonylheptyl) bicyclo [4.4.0] deca-2,5,7-triene	C_25_H_40_O_2_	372	0.67
35	36.60	Hexadecenoic acid,1-(hydroxymethyl)-1,2-ethanediyl ester	C_35_H_68_O_5_	568	4.55
36	36.76	Hexadecanoic acid, 2,3-dihydroxypropylester	C_24_H_38_O_4_	390	3.36
37	39.46	Ethyl iso-allocholate	C_26_H_44_O_5_	436	2.09
39	39.76	7,8-Epoxylanostan-11-ol, 3-acetoxy-	C_32_H_54_O_4_	502	3.29

RT: relation time, MF: molecular formula, MW: molecular weight.

**Table 4:** Bioactivity of major compounds in the RCFE.

NO.	Chemical Name	Classification	Reported Activity **
1	2-Methylcyclopentanone	Ketones	Anti-inflammatory, analgesic, anticonvulsant, and antibacterial
2	Cyclohexanamine, N-3-butenyl-N-methyl-	Alkaloid	No reporter activity
3	4H-Pyran-4-one, 2,3-dihydro-3,5-dihydroxy-6-methyl	Ketone	Antimicrobial and anti-inflammatory
4	5-Hydroxymethylfurfural	Ketone	Anti-allergenic, anti-diabetic, anti-inflammatory, and antimicrobial
5	Glycerol 1,2-diacetate	Glycerolipids	Flavoring agent and food additive
6	Maltose	Sugar	Antioxidant, antidiabetic, and anti-inflammatory activity
7	Hexadecanoic acid	Fatty acid	Anti-inflammatory, hypocholesterolemia, and antioxidant
8	9,12-Octadecadienoyl chloride, (Z, Z)-	Fatty acid	Hypocholesterolemia, hepatoprotective, antiandrogenic, antihistaminic, and anticancer
9	Oleic Acid	Fatty acid	Antibacterial, anticancer, antiandrogenic, and dermatogenic activities
10	Hexadecenoic acid, 1-(hydroxymethyl)-1,2-ethanediyl ester	Fatty acid derivative	Antioxidant, antiandrogenic, and hypocholesterolemia
11	Hexadecanoic acid, 2,3-dihydroxypropyl ester	Fatty acid derivative	Antibacterial activity
12	Ethyl iso-allocholate	Alkaloid	Anti-inflammatory, anticancer, antimicrobial, antiasthma, and diuretic
13	7,8-Epoxylanostan-11-ol, 3-acetoxy-	Alcoholic compound	Anti-inflammatory and antimicrobial

** Dr. Duke’s Phytochemical and Ethnobotanical Databases.

**Table 5:** HPLC analysis results of the RCFE.

Components	RT (min)	Conc. (μg/mg)
Flavonoid compounds		
Naringin	4.4	4.01
Rutin	5.2	14.23
Quercetin	7.0	4.40
Kaempferol	8.0	3.89
Luteolin	9.0	6.65
Phenolic compounds		
Chlorogenic acid	3.0	4.55
Syringenic acid	5.0	1.88
*p*-Coumaric acid	6.0	2.14
Cinnamic acid	7.0	10.44
Caffeic acid	8.1	2.03
Pyrogallol	9.2	0.56
Gallic acid	9.8	13.45
Ferulic acid	10.8	5.14

**Table 6:** Changes in pH values of the mayonnaise samples enriched with the RCFE during storage.

	0 Time	15 Days	30 Days	45 Days	60 Days
C1	4.45 ^a^ ± 0.07	4.03 ^c^ ± 0.05	3.63 ^d^ ± 0.01	3.10 ^d^ ± 0.10	2.76 ^d^ ± 0.11
C2	4.45 ^a^ ± 0.06	4.30 ^a^ ± 0.01	4.25 ^a^ ± 0.02	3.99 ^bc^ ± 0.03	3.85 ^bc^ ± 0.02
T1	4.35 ^b^ ± 0.04	4.29 ^a^ ± 0.01	4.11 ^c^ ± 0.02	3.93 ^c^ ± 0.04	3.79 ^c^ ± 0.01
T2	4.35 ^b^ ± 0.01	4.29 ^a^ ± 0.01	4.21 ^b^ ± 0.01	4.03 ^b^ ± 0.05	3.89 ^ab^ ± 0.02
T3	4.27 ^bc^ ± 0.07	4.23 ^b^ ± 0.02	4.20 ^b^ ± 0.02	4.14 ^a^ ± 0.02	3.91 ^ab^ ± 0.01
T4	4.20 ^c^ ± 0.02	4.19 ^b^ ± 0.01	4.20 ^b^ ± 0.04	4.15 ^a^ ± 0.03	3.96 ^a^ ± 0.02

Means with the same letter in columns are not statistically significant (*p* ≤ 0.05), while means sharing a different letter in columns of the table are significantly statistical (*p* ≤ 0.05). C1, mayonnaise with no antioxidants; C2, mayonnaise containing 0.02% BHT; T1, T2, T3, T4: mayonnaise containing 0.125, 0.25, 0.5, and 0.75% ethanolic *Rosa canina* extract, respectively.

**Table 7:** Changes in the acid value (mg KOH g^1^) of mayonnaise samples enriched with RCFE during storage.

	0 Time	15 Days	30 Days	45 Days	60 Days
C1	0.80 ^a^ ± 0.01	2.63 ^a^ ± 0.09	3.25 ^a^ ± 0.07	4.06 ^a^ ± 0.11	6.54 ^a^ ± 0.05
C2	0.71 ^b^ ± 0.02	1.21 ^c^ ± 0.04	1.73 ^c^ ± 0.05	2.14 ^c^ ± 0.06	3.17 ^c^ ± 0.08
T1	0.74 ^b^ ± 0.01	1.39 ^b^ ± 0.04	1.89 ^b^ ± 0.08	2.57 ^b^ ± 0.36	3.72 ^b^ ± 0.04
T2	0.73 ^b^ ± 0.03	1.37 ^b^ ± 0.03	1.72 ^c^ ± 0.04	2.22 ^c^ ± 0.04	3.17 ^c^ ± 0.07
T3	0.72 ^b^ ± 0.03	1.30 ^bc^ ± 0.01	1.73 ^c^ ± 0.04	2.01 ^c^ ± 0.04	3.03 ^d^ ± 0.01
T4	0.74 ^b^ ± 0.04	1.24 ^c^ ± 0.04	1.63 ^c^ ± 0.07	1.99 ^c^ ± 0.01	2.99 ^d^ ± 0.02

Means with the same letter in columns are not statistically significant (*p* ≤ 0.05), while means sharing a different letter in columns of the table are significantly statistical (*p* ≤ 0.05). C1, mayonnaise with no antioxidants; C2, mayonnaise containing 0.02% BHT; T1, T2, T3, T4: mayonnaise containing 0.125, 0.25, 0.5, and 0.75% ethanolic *Rosa canina* extract, respectively.

**Table 8:** Changes in peroxide value of mayonnaise samples enriched with RCFE during storage.

	0 Time	15 Days	30 Days	45 Days	60 Days
C1	0.56 ^a^ ± 0.01	6.24 ^a^ ± 0.09	9.10 ^a^ ± 0.20	15.01 ^a^ ± 0.50	17.26 ^a^ ± 0.40
C2	0.51 ^d^ ± 0.02	2.67 ^d^ ± 0.07	6.49 ^c^ ± 0.05	7.56 ^c^ ± 0.18	8.57 ^c^ ± 0.27
T1	0.55 ^ab^ ± 0.01	3.16 ^b^ ± 0.03	7.84 ^b^ ± 0.09	9.13 ^b^ ± 0.05	10.47 ^b^ ± 0.07
T2	0.53 ^bcd^ ± 0.01	3.09 ^c^ ± 0.04	6.76 ^c^ ± 0.09	7.04 ^d^ ± 0.12	8.11 ^d^ ± 0.27
T3	0.54 ^bc^ ± 0.03	3.03 ^c^ ± 0.07	6.66 ^c^ ± 0.04	6.79 ^d^ ± 0.05	6.96 ^e^ ± 0.12
T4	0.52 ^cd^ ± 0.01	3.03 ^c^ ± 0.07	3.99 ^d^ ± 0.03	4.46 ^e^ ± 0.10	4.74 ^f^ ± 0.10

Means with the same letter in columns are not statistically significant (*p* ≤ 0.05), while means sharing a different letter in columns of the table are significantly statistical (*p* ≤ 0.05). C1, mayonnaise with no antioxidants; C2, mayonnaise containing 0.02% BHT; T1, T2, T3, T4: mayonnaise containing 0.125, 0.25, 0.5, and 0.75% ethanolic *Rosa canina* extract, respectively.

**Table 9:** TBARS values (mg MAD kg^−1^ sample) of mayonnaise samples enriched with RCFE during storage.

	0 Time	15 Days	30 Days	45 Days	60 Days
C1	0.424 ^a^ ± 0.005	0.626 ^a^ ± 0.004	0.930 ^a^ ± 0.031	1.123 ^a^ ± 0.105	1.516 ^a^ ± 0.020
C2	0.430 ^a^ ± 0.005	0.473 ^c^ ± 0.019	0.561 ^c^ ± 0.002	0.644 ^c^ ± 0.004	0.722 ^d^ ± 0.008
T1	0.414 ^a^ ± 0.006	0.510 ^b^ ± 0.005	0.628 ^b^ ± 0.006	0.744 ^b^ ± 0.007	0.837 ^b^ ± 0.005
T2	0.436 ^a^ ± 0.014	0.493 ^b^ ± 0.007	0.601 ^b^ ± 0.007	0.734 ^b^ ± 0.005	0.768 ^c^ ± 0.027
T3	0.438 ^a^ ± 0.027	0.461 ^c^ ± 0.004	0.542 ^c^ ± 0.011	0.625 ^c^ ± 0.019	0.676 ^e^ ± 0.015
T4	0.426 ^a^ ± 0.006	0.439 ^d^ ± 0.015	0.533 ^c^ ± 0.040	0.532 ^d^ ± 0.050	0.631 ^f^ ± 0.010

Means with the same letter in columns are not statistically significant (*p* ≤ 0.05), while means sharing a different letter in columns of the table are significantly statistical (*p* ≤ 0.05). C1, mayonnaise with no antioxidants; C2, mayonnaise containing 0.02% BHT; T1, T2, T3, T4: mayonnaise containing 0.125, 0.25, 0.5, and 0.75% ethanolic *Rosa canina* extract, respectively.

**Table 10:** Means of taste and color values of mayonnaise samples enriched with RCFE during storage.

	Color					Taste				
	0 Time	15 Days	30 Days	45 Days	60 Days	0 Time	15 Days	30 Days	45 Days	60 Days
C1	8.46 ^a^ ± 0.53	7.54 ^d^ ± 0.27	5.89 ^e^ ± 0.53	5.23 ^d^ ± 0.57	4.01 ^d^ ± 0.52	8.14 ^a^ ± 0.43	6.26 ^c^ ± 0.23	6.00 ^d^ ± 0.80	5.32 ^c^ ± 0.46	3.14 ^c^ ± 0.56
C2	8.46 ^a^ ± 0.48	8.05 ^a^ ± 0.63	8.20 ^a^ ± 0.62	6.74b ^c^ ± 0.35	6.08 ^bc^ ± 0.43	8.15 ^a^ ± 0.25	7.74 ^a^ ± 0.25	7.64 ^a^ ± 0.23	6.98 ^bc^ ± 0.58	5.77 ^a^ ± 0.38
T1	8.27 ^b^ ± 0.65	7.75 ^c^ ± 0.52	7.19 ^d^ ± 0.73	6.64 ^c^ ± 0.75	5.98 ^c^ ± 0.25	7.98 ^ab^ ± 0.32	7.53 ^b^ ± 0.54	7.40 ^c^ ± 0.45	6.88 ^c^ ± 0.32	5.4 ^b^ ± 0.57
T2	8.27 ^b^ ± 0.82	8.02 ^a^ ± 0.57	7.36 ^c^ ± 0.43	6.82 ^b^ ± 0.45	6.14 ^ab^ ± 0.52	7.96 ^ab^ ± 0.40	7.72 ^a^ ± 0.42	7.57 ^ab^ ± 0.75	7.06 ^b^ ± 0.83	5.83 ^a^ ± 0.25
T3	8.12 ^bc^ ± 0.74	7.91 ^b^ ± 0.43	7.85 ^b^ ± 0.32	7.00 ^a^ ± 0.80	6.17 ^ab^ ± 0.46	7.82 ^bc^ ± 0.35	7.61 ^ab^ ± 0.53	7.55 ^b^ ± 1.10	7.25 ^a^ ± 0.52	5.86 ^a^ ± 0.32
T4	7.98 ^c^ ± 0.81	7.83 ^bc^ ± 0.37	7.86 ^b^ ± 0.25	7.01 ^a^ ± 0.80	6.25 ^a^ ± 0.34	7.72 ^c^ ± 1.35	7.54 ^b^ ± 0.81	7.56 ^ab^ ± 0.72	7.26 ^a^ ± 0.42	5.93 ^a^ ± 0.25

Means with the same letter in columns are not statistically significant (*p* ≤ 0.05), while means sharing a different letter in columns of the table are significantly statistical (*p* ≤ 0.05). C1, mayonnaise with no antioxidants; C2, mayonnaise treated by 0.02% BHT; T1, T2, T3, T4: mayonnaise treated by 0.125, 0.25, 0.5, and 0.75% ethanolic *Rosa canina* extract, respectively.

**Table 11:** Means of odor and mouthfeel values of mayonnaise samples enriched with RCFE during storage.

	Odor					Mouthfeel				
	0 Time	15 Days	30 Days	45 Days	60 Days	0 Time	15 Days	30 Days	45 Days	60 Days
C1	8.37 ^a^ ± 0.57	7.58 ^c^ ± 0.81	5.90 ^d^ ± 0.28	5.00 ^d^ ± 0.75	3.26 ^e^ ± 0.35	8.69 ^a^ ± 0.35	7.30 ^c^ ± 0.83	7.15 ^c^ ± 0.65	4.42 ^d^ ± 0.37	3.53 ^d^ ± 0.29
C2	8.37 ^a^ ± 0.57	8.09 ^a^ ± 0.57	7.99 ^a^ ± 0.53	7.50 ^b^ ± 0.63	7.23 ^a^ ± 1.10	8.68 ^a^ ± 0.51	7.79 ^a^ ± 0.75	7.35 ^b^ ± 0.72	6.57 ^b^ ± 0.44	6.16 ^b^ ± 0.45
T1	8.19 ^b^ ± 0.42	8.07 ^a^ ± 0.65	7.73 ^c^ ± 0.49	6.40 ^c^ ± 0.27	5.13 ^c^ ± 0.75	8.49 ^b^ ± 0.47	7.77 ^a^ ± 0.57	7.11 ^c^ ± 0.52	6.06 ^c^ ± 0.53	5.20 ^c^ ± 0.64
T2	8.18 ^b^ ± 0.35	8.06 ^a^ ± 0.54	7.91 ^b^ ± 0.53	7.58 ^b^ ± 0.45	6.23 ^c^ ± 0.82	8.48 ^b^ ± 0.21	7.76 ^a^ ± 0.37	7.28 ^b^ ± 0.48	6.66 ^ab^ ± 0.75	6.22 ^ab^ ± 0.35
T3	8.04 ^bc^ ± 0.53	7.95 ^b^ ± 0.43	7.89 ^b^ ± 0.75	7.79 ^a^ ± 0.57	7.35 ^a^ ± 0.81	8.33 ^bc^ ± 0.73	7.65 ^b^ ± 0.28	7.60 ^a^ ± 0.23	6.84 ^a^ ± 0.57	6.25 ^ab^ ± 0.52
T4	7.89 ^c^ ± 0.58	7.87 ^b^ ± 0.35	7.90 ^b^ ± 0.37	7.80 ^a^ ± 0.25	6.43 ^b^ ± 0.53	8.19 ^c^ ± 1.02	7.58 ^b^ ± 0.53	7.27 ^b^ ± 0.42	6.85 ^a^ ± 0.43	6.33 ^a^ ± 0.81

Means with the same letter in columns are not statistically significant (*p* ≤ 0.05), while means sharing a different letter in columns of the table are significantly statistical (*p* ≤ 0.05). C1, mayonnaise with no antioxidants; C2, mayonnaise treated by 0.02% BHT; T1, T2, T3, T4: mayonnaise treated by 0.125, 0.25, 0.5, and 0.75% ethanolic *Rosa canina* extract, respectively.

**Table 12:** Means of overall acceptability values of mayonnaise samples enriched with RCFE during storage.

	0 Time	15 Days	30 Days	45 Days	60 Days
C1	8.10 ^a^ ± 0.25	6.94 ^c^ ± 0.72	6.33 ^d^ ± 0.27	4.03 ^d^ ± 0.47	3.04 ^d^ ± 0.52
C2	8.11 ^a^ ± 0.31	7.40 ^a^ ± 0.63	7.31 ^a^ ± 0.38	5.18 ^bc^ ± 0.43	4.23 ^bc^ ± 0.47
T1	7.92 ^b^ ± 0.45	7.39 ^a^ ± 0.58	7.07 ^c^ ± 0.54	5.11 ^c^ ± 0.32	4.16 ^c^ ± 0.35
T2	7.92 ^b^ ± 0.60	7.37 ^a^ ± 0.47	7.24 ^b^ ± 0.40	5.24 ^b^ ± 0.32	4.27 ^ab^ ± 0.23
T3	7.78 ^bc^ ± 0.52	7.27 ^b^ ± 0.52	7.22 ^b^ ± 0.39	5.39 ^a^ ± 0.27	4.30 ^ab^ ± 0.27
T4	7.64 ^c^ ± 0.23	7.20 ^b^ ± 0.37	7.22 ^b^ ± 0.33	5.39 ^a^ ± 0.29	4.35 ^a^ ± 0.25

Means with the same letter in columns are not statistically significant (*p* ≤ 0.05), while means sharing a different letter in columns of the table are significantly statistical (*p* ≤ 0.05). C1, mayonnaise with no antioxidants; C2, mayonnaise treated by 0.02% BHT; T1, T2, T3, T4: mayonnaise treated by 0.125, 0.25, 0.5, and 0.75% ethanolic *Rosa canina* extract, respectively.

**Table 13:** Changes in mayonnaise samples’ L*, a*, and b* values enriched with RCFE during storage.

	L*					a*					b*				
	0 Time	15 Days	30 Days	45 Days	60 Days	0 Time	15 Days	30 Days	45 Days	60 Days	0 Time	15 Days	30 Days	45 Days	60 Days
C1	76.73 ^a^ ± 0.38	71.33 ^b^ ± 1.11	68.57 ^b^ ± 0.40	49.97 ^d^ ± 1.59	42.53 ^c^ ± 2.40	−8.47 ^d^ ± 0.64	−7.20 ^d^ ± 0.30	−4.90 ^d^ ± 0.26	−3.70 ^d^ ± 0.46	−2.77 ^cd^ ± 0.70	42.27 ^a^ ± 1.53	38.13 ^b^ ± 1.11	36.30 ^a^ ± 0.30	32.00 ^b^ ± 0.46	25.87 ^de^ ± 0.74
C2	75.27 ^a^ ± 0.21	74.20 ^a^ ± 1.05	71.20 ^a^ ± 1.08	66.20 ^a^ ± 0.95	64.83 ^a^ ± 0.55	−8.73 ^d^ ± 0.15	−8.03 ^e^ ± 0.15	−7.23 ^e^ ± 0.42	−5.63 ^e^ ± 0.40	−4.87 ^e^ ± 0.15	43.60 ^a^ ± 1.10	40.03 ^a^ ± 0.50	36.67 ^a^ ± 0.15	35.73 ^a^ ± 0.35	34.60 ^a^ ± 0.36
T1	71.67 ^b^ ± 1.56	69.03 ^c^ ± 0.31	66.90 ^c^ ± 0.56	65.80 ^a^ ± 0.36	58.70 ^b^ ± 0.53	−6.30 ^c^ ± 0.20	−5.07 ^c^ ± 0.15	−4.57 ^d^ ± 0.32	−4.07 ^d^ ± 0.25	−3.23 ^d^ ± 0.90	39.87 ^b^ ± 0.90	37.30 ^b^ ± 0.61	36.03 ^a^ ± 0.15	34.80 ^a^ ± 0.46	29.00 ^b^ ± 0.44
T2	67.67 ^c^ ± 0.76	62.97 ^d^ ± 0.91	61.83 ^d^ ± 1.08	59.77 ^b^ ± 1.40	57.57 ^b^ ± 0.61	−6.27 ^c^ ± 0.55	−4.20 ^b^ ± 0.66	−2.60 ^c^ ± 0.56	−2.40 ^c^ ± 0.36	−1.83 ^c^ ± 0.47	36.20 ^c^ ± 0.30	34.53 ^c^ ± 0.59	33.73 ^b^ ± 0.67	32.57 ^b^ ± 1.07	27.20 ^c^ ± 0.79
T3	64.10 ^d^ ± 1.57	59.97 ^e^ ± 1.30	60.37 ^e^ ± 0.06	59.13 ^b^ ± 0.65	57.37 ^b^ ± 0.55	−4.13 ^b^ ± 0.59	−2.57 ^a^ ± 0.31	−1.83 ^b^ ± 0.25	−0.93 ^b^ ± 0.40	0.33 ^b^ ± 0.15	34.70 ^c^ ± 0.53	33.50 ^c^ ± 0.40	31.37 ^c^ ± 0.45	29.03 ^c^ ± 0.70	26.80 ^cd^ ± 0.66
T4	64.20 ^d^ ± 0.87	59.77 ^e^ ± 0.32	58.03 ^f^ ± 0.71	57.20 ^c^ ± 1.01	57.03 ^b^ ± 0.81	−2.30 ^a^ ± 1.73	−2.80 ^a^ ± 0.26	−1.10 ^a^ ± 0.20	0.30 ^a^ ± 0.10	1.43 ^a^ ± 0.75	32.80 ^d^ ± 0.53	31.50 ^d^ ± 0.79	30.20 ^d^ ± 0.44	26.97 ^d^ ± 0.45	25.07 ^e^ ± 0.40

Means with the same letter in columns are not statistically significant (*p* ≤ 0.05), while means sharing a different letter in columns of the table are significantly statistical (*p* ≤ 0.05). C1, mayonnaise with no antioxidants; C2, mayonnaise treated by 0.02% BHT; T1, T2, T3, T4: mayonnaise treated by 0.125, 0.25, 0.5, and 0.75% ethanolic *Rosa canina* extract, respectively.

**Table 14:** Changes in ΔE and chroma values of mayonnaise samples enriched with RCFE during storage.

	ΔE					Chroma				
	0 Time	15 Days	30 Days	45 Days	60 Days	0 Time	15 Days	30 Days	45 Days	60 Days
C1	0.00 ^e^ ± 0.00	0.00 ^d^ ± 0.00	0.00 ^f^ ± 0.00	0.00 ^c^ ± 0.00	0.00 ^d^ ± 0.00	43.11 ^a^ ± 1.55	38.81 ^b^ ± 1.12	36.63 ^b^ ± 0.33	32.22 ^c^ ± 0.43	26.02 ^cd^ ± 0.67
C2	2.10 ^d^ ± 0.80	3.64 ^c^ ± 0.52	3.56 ^d^ ± 1.07	16.78 ^a^ ± 0.83	24.04 ^a^ ± 0.51	44.47 ^a^ ± 1.07	40.83 ^a^ ± 0.52	37.38 ^a^ ± 0.09	36.18 ^a^ ± 0.38	34.94 ^a^ ± 0.38
T1	6.04 ^c^ ± 1.68	3.29 ^c^ ± 0.28	1.74 ^e^ ± 0.57	16.09 ^a^ ± 0.42	16.49 ^b^ ± 0.59	40.36 ^b^ ± 0.91	37.64 ^b^ ± 0.59	36.32 ^b^ ± 0.17	35.04 ^b^ ± 0.44	29.19 ^bc^ ± 0.41
T2	11.15 ^b^ ± 0.58	9.59 ^b^ ± 1.21	7.61 ^c^ ± 0.88	9.95 ^b^ ± 1.34	15.14 ^c^ ± 0.58	36.74 ^c^ ± 0.32	34.79 ^c^ ± 0.66	33.84 ^c^ ± 0.62	32.66 ^c^ ± 1.07	27.26 ^b^ ± 0.77
T3	15.38 ^a^ ± 1.32	13.16 ^a^ ± 1.30	10.05 ^b^ ± 0.31	10.05 ^b^ ± 0.64	15.19 ^c^ ± 0.51	34.95 ^d^ ± 0.46	33.50 ^d^ ± 0.41	31.42 ^d^ ± 0.46	29.05 ^d^ ± 0.70	26.80 ^bcd^ ± 0.66
T4	16.95 ^a^ ± 0.29	14.06 ^a^ ± 0.13	12.76 ^a^ ± 0.73	9.70 ^b^ ± 0.73	15.14 ^c^ ± 0.60	32.91 ^e^ ± 0.48	31.62 ^e^ ± 0.79	30.22 ^e^ ± 0.44	26.97 ^e^ ± 0.45	25.11 ^d^ ± 0.41

Means with the same letter in columns are not statistically significant (*p* ≤ 0.05), while means sharing a different letter in columns of the table are significantly statistical (*p* ≤ 0.05). C1, mayonnaise with no antioxidants; C2, mayonnaise treated by 0.02% BHT; T1, T2, T3, T4: mayonnaise treated by 0.125, 0.25, 0.5, and 0.75% ethanolic *Rosa canina* extract, respectively.

## Data Availability

The data used to support the findings of this study are included within the article.
